# Critical aspects of using bacterial cell viability assays with the fluorophores SYTO9 and propidium iodide

**DOI:** 10.1186/s12866-015-0376-x

**Published:** 2015-02-18

**Authors:** Philipp Stiefel, Sabrina Schmidt-Emrich, Katharina Maniura-Weber, Qun Ren

**Affiliations:** Laboratory for Biointerfaces, Swiss Federal Laboratories for Materials Science and Technology (Empa), Lerchenfeldstrasse 5, CH-9014 St. Gallen, Switzerland

**Keywords:** SYTO9, Propidium iodide, Viability staining, Bacterial live/dead cells

## Abstract

**Background:**

Viability staining with SYTO9 and propidium iodide (PI) is a frequently used tool in microbiological studies. However, data generated by such routinely used method are often not critically evaluated for their accuracy. In this study we aim to investigate the critical aspects of this staining method using *Staphylococcus aureus* and *Pseudomonas aeruginosa* as the model microorganisms for high throughput studies in microtiter plates. SYTO9 or PI was added alone or consecutively together to cells and the fluorescence intensities were measured using microplate reader and confocal laser scanning microscope.

**Results:**

We found that staining of *S. aureus* cells with SYTO9 alone resulted in equal signal intensity for both live and dead cells, whereas staining of *P. aeruginosa* cells led to 18-fold stronger signal strength for dead cells than for live ones. After counterstaining with PI, the dead *P. aeruginosa* cells still exhibited stronger SYTO9 signal than the live cells. We also observed that SYTO9 signal showed strong bleaching effect and decreased dramatically over time. PI intensity of the culture increased linearly with the increase of dead cell numbers, however, the maximum intensities were rather weak compared to SYTO9 and background values. Thus, slight inaccuracy in measurement of PI signal could have significant effect on the outcome.

**Conclusions:**

When viability staining with SYTO9 and PI is performed, several factors need to be considered such as the bleaching effect of SYTO9, different binding affinity of SYTO9 to live and dead cells and background fluorescence.

**Electronic supplementary material:**

The online version of this article (doi:10.1186/s12866-015-0376-x) contains supplementary material, which is available to authorized users.

## Background

Bacterial viability assays are widely used for example to evaluate antimicrobial properties, to perform microbiological quality monitoring of water, and to determine the viability of unculturable environmental species. They have proven values in areas such as medicine, biotechnology, food industry, as well as environmental monitoring to assess the susceptibility of bacteria against biocides.

The most used techniques to assess bacterial viability are based on indirect measurements of the state of the cells, without any direct indication that bacteria are capable of growth and division. These methods focus on nucleic acid stains, membrane potential, redox indicators, or reporter gene systems (Reviewed in [1]). There have been different opinions on the criteria for bacterial viability to define a bacterial cell as dead or alive [2-4]. Cellular and membrane integrity is considered to be one criterion distinguishing between viable and dead bacterial cells [5]. Viable cells are assumed to have intact and tight cell membranes that cannot be penetrated by some staining compounds, whereas dead cells are considered to have disrupted and/or broken membranes. However, situations could occur where cells maintain membrane integrity, but are metabolically inactive [4]. In contrary, there are conditions where membrane integrity of viable cells is reduced such as during fast exponential growth in nutrient rich environments [6]. Thus, external medium or environment and the physiological status of the cells can influence the viability staining [6]. These influences can result in an under- or overestimation of the number of viable cells and may lead to incorrect conclusions.

Bacterial viability tests are often performed with premixed, ready for use, dual staining kits, such as the *Bac*Light™ (Live/Dead Bacterial Viability Kit, L-7007, Molecular Probes, [7,8]), composed of two fluorophores SYTO9 and propidium iodide (PI) based on the detection of membrane integrity. Advantages of using such a kit are a rapid procedure, quantitative analyses, as well as the possibility to measure using various instruments such as flow cytometer [8-10], microplate reader [11,12], and microscope [13,14]. The risk to employ a premixed kit of SYTO9/PI is, amongst others, the lack of possibility to monitor and subtract all respective background signals.

The red-fluorescent nucleic acid stain PI intercalates to DNA with no sequence preference with one dye molecule per four to five base pairs, similar to ethidium bromide [15]. When bound to DNA fluorescence of PI is enhanced 20- to 30-fold [16]. PI is commonly used for identifying dead cells in a population and as counterstain in multicolor fluorescent techniques because it is supposed to penetrate only cells with disrupted membranes and is generally excluded from viable cells. In contrary, the green-fluorescent nucleic acid stain SYTO9 enters live and dead bacterial cells. The fluorescent signal of SYTO9 is strongly enhanced when bound to nucleic acid and shows low intrinsic fluorescence signal when unbound. When both dyes are present, PI exhibits a stronger affinity for nucleic acids than SYTO9, and hence, SYTO9 is displaced by PI [17]. Stocks *et al.* determined the association constants of PI at 3.7 × 10^5^/M and SYTO9 at 1.8 × 10^5^/M [17].

The aim of this work was to evaluate to which extent viability assays (live/dead staining) can be applied as routine technique and to which extent validation is required, as this method is widely used in various research areas, and applied to various instruments. We identified and studied the critical aspects of the SYTO9/PI staining using *Staphylococcus aureus* and *Pseudomonas aeruginosa* as the model microorganisms and based on the data acquired from microplate reader. The samples were further studied by fluorescence microscopy for quantitative and qualitative analysis.

## Results

### Approach to obtain live and dead bacterial cells

To obtain dead cells isopropanol was first tested. It has been reported that isopropanol increases permeability of the bacterial cell membrane and destroys protein function by denaturing them, thereby kills bacteria [18,19].

Bacterial cells from the same pre-culture were either treated with 70% isopropanol or with 0.9% NaCl solution. Isopropanol treated cells led to no colonies in the agar plating experiments, whereas expected number of colonies was obtained from the NaCl treated cells (data not shown). Therefore, cells treated with isopropanol were referred as dead cells in this study and NaCl solution treated cells as live cells. Furthermore, the differently treated cells exhibited similar values (with less than 10% difference) of optical density (OD). This result suggests that the cells, even if dead, kept structural integrity after the treatment with isopropanol. This suggestion was further supported by observation of similar numbers of green-colored cells (live) and red/yellow-colored cells (dead) with similar shape under the microscope (Additional file 1: Figure S1). Therefore, the accordingly treated cells were further used as live and dead cells for the staining tests.

### SYTO9 staining

**Figure 1:**
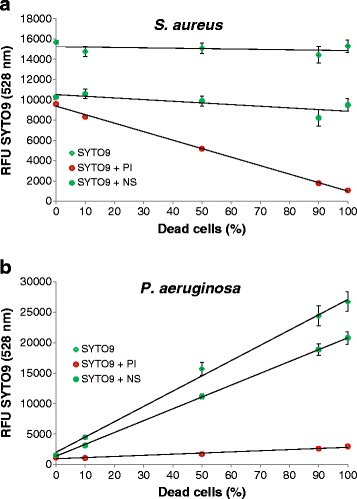
**SYTO9 staining analyzed with microplate reader.** Relative fluorescence intensity (RFU) at 528 nm is shown for different live/dead proportions of *S. aureus* **(a)** and *P. aeruginosa* **(b)**. Values were measured after staining with SYTO9 for 15 minutes (green diamonds) and after additional 15 minutes counterstaining with PI (red circles). As a control 0.9% NaCl solution (NS) was added (green circles) instead of PI to consider dilution and bleaching effects of SYTO9. Cell optical densities (OD) of 0.25 for *S. aureus* and 0.12 for *P. aeruginosa* were used. Error bars represent 3 individual repeats with 3 replicas for *P. aeruginosa* and *S. aureus*, respectively. The error bars for some data points are too small to be seen (hidden behind the symbols).

Mixtures of different ratios of live and dead cells were stained with SYTO9 alone. The fluorescence intensity was measured with the microplate reader. As expected for staining with a membrane permeable dye like SYTO9, no difference in intensity was observed between live and dead cells of *S. aureus* (Figure 1a). However, for *P. aeruginosa* with the same total cell numbers 100% dead cells exhibited an 18-fold stronger signal than 100% live cells (Figure 1b). This finding is further supported by the intermediate signal intensity of the different mixtures, showing a linear increase with the increase of the fraction of dead cells. The effect of stronger SYTO9 staining of dead cells seems to be common for Gram-negative bacteria as we observed the same effect for *Escherichia coli*, but not for the Gram-positive *Bacillus subtilis* (data not shown).

### SYTO9 signal after counterstaining with PI

To distinguish live cells from dead ones, PI was added to the mixtures having different live/dead ratios of SYTO9 stained cells. A clear reduction in SYTO9 staining was observed for the dead cells of both strains compared to control samples which were treated with NaCl solution (Figure 1). For 100% dead cells of *S. aureus* and *P. aeruginosa* the fluorescence intensity of SYTO9 was decreased 87% and 85%, respectively, compared to the control samples based on the measurement with the microplate reader. On the contrary, living cells were significantly less de-stained by the addition of PI, e.g. 5% reduction in SYTO9 signal for 100% *S. aureus* live cells and 20% for *P. aeruginosa*. Thus, the dead cells of *S. aureus* exhibited 9-fold weaker SYTO9 signal intensity than the living cells, whereas the dead cells of *P. aeruginosa* still displayed 2.7-fold higher SYTO9 intensity than the living ones after counterstaining with PI (Figure 1). These results demonstrate that the displacement of SYTO9 by PI takes place as expected. However, in *P. aeruginosa* even if the dead cells show strong reduction in SYTO9 fluorescence after PI counterstaining, they possess still stronger SYTO9 fluorescence than the living ones. Living cells showed no or only slight reduction in SYTO9 fluorescence after counterstaining, which is expected because PI should not enter intact cells to replace SYTO9.

**Figure 2:**
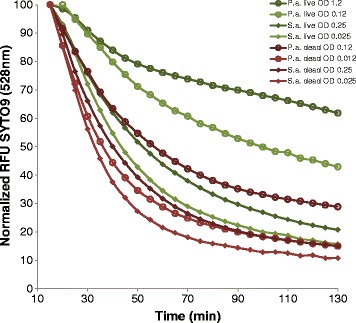
**Bleaching of SYTO9 over time.** Different amounts of live or dead cells of *S. aureus* (S.a.) and *P. aeruginosa* (P.a.) were stained with SYTO9, respectively. After 15 min incubation fluorescence intensity at 528 nm was automatically measured every 5 minutes with the microplate reader. Starting RFU value was set to 100% which was used to normalize other values.

During the experiments strong reduction of SYTO9 fluorescence with time was observed, which indicates that SYTO9 is prone for bleaching. Therefore, the possibility of SYTO9 bleaching was investigated by measuring green fluorescence of SYTO9 stained cells every 5 minutes. About 4-8% of the SYTO9 signal intensity was lost every 5 minutes, depending on the physiological state of the cell and cell number (Figure 2 relative values, Additional file 2: Figure S2 absolute values). Different trends can be observed. First, the reduction rate of SYTO9 signal decreases with higher cell numbers. Second, the reduction rate is higher for the same amount of dead compared to live cells. Interestingly, particular differences in bleaching were observed for live *P. aeruginosa* cells, which were also shown to be difficult to stain (Figure 1).

### PI signal after counterstaining

**Figure 3:**
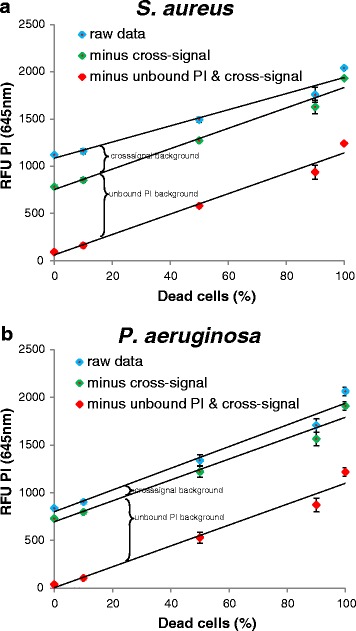
**Propidium iodide (PI) staining measured with microplate reader.** Relative fluorescence intensity at 645 nm is shown for different live/dead proportions of *S. aureus* **(a)** and *P. aeruginosa* **(b)**. Values were measured using the microplate reader after 15 minutes staining with PI of SYTO9 pre-stained cells. OD values of 0.25 for *S. aureus* and 0.12 for *P. aeruginosa* were used. Error bars represent 3 individual repeats with 3 replicas. The error bars for some data points are too small to be seen (hidden behind the symbols). Blue diamonds: mean values of the raw data; green diamonds: calculated values after subtraction SYTO9 cross-signal at 645 nm; red diamonds: values after additionally subtracting background of unbound PI.

Upon counterstaining, the PI signal in living *S. aureus* cells was almost zero after subtraction of background signals (cross-signal of SYTO9 and unbound PI signal), as expected for this membrane-impermeable dye. With increased proportion of dead to live cells the red PI fluorescence increased linearly (Figure 3). However, the absolute fluorescent intensity value was rather low. Unbound PI possessed strong background signal with a relative fluorescence intensity unit (RFU) of about 700. The dead cells exhibited a RFU of 1200 after the background subtraction (cross-signal of SYTO9 and unbound PI signal). The background signals of unbound PI could not be prevented in fluorescence readouts. Therefore, for reliable interpretation of the PI fluorescence data obtained from the microplate reader background controls and relatively high numbers of dead cells are needed. Precise quantitative determination of the amount of dead cells is hence rather difficult.

### Microscopical examination of live/dead staining

**Figure 4:**
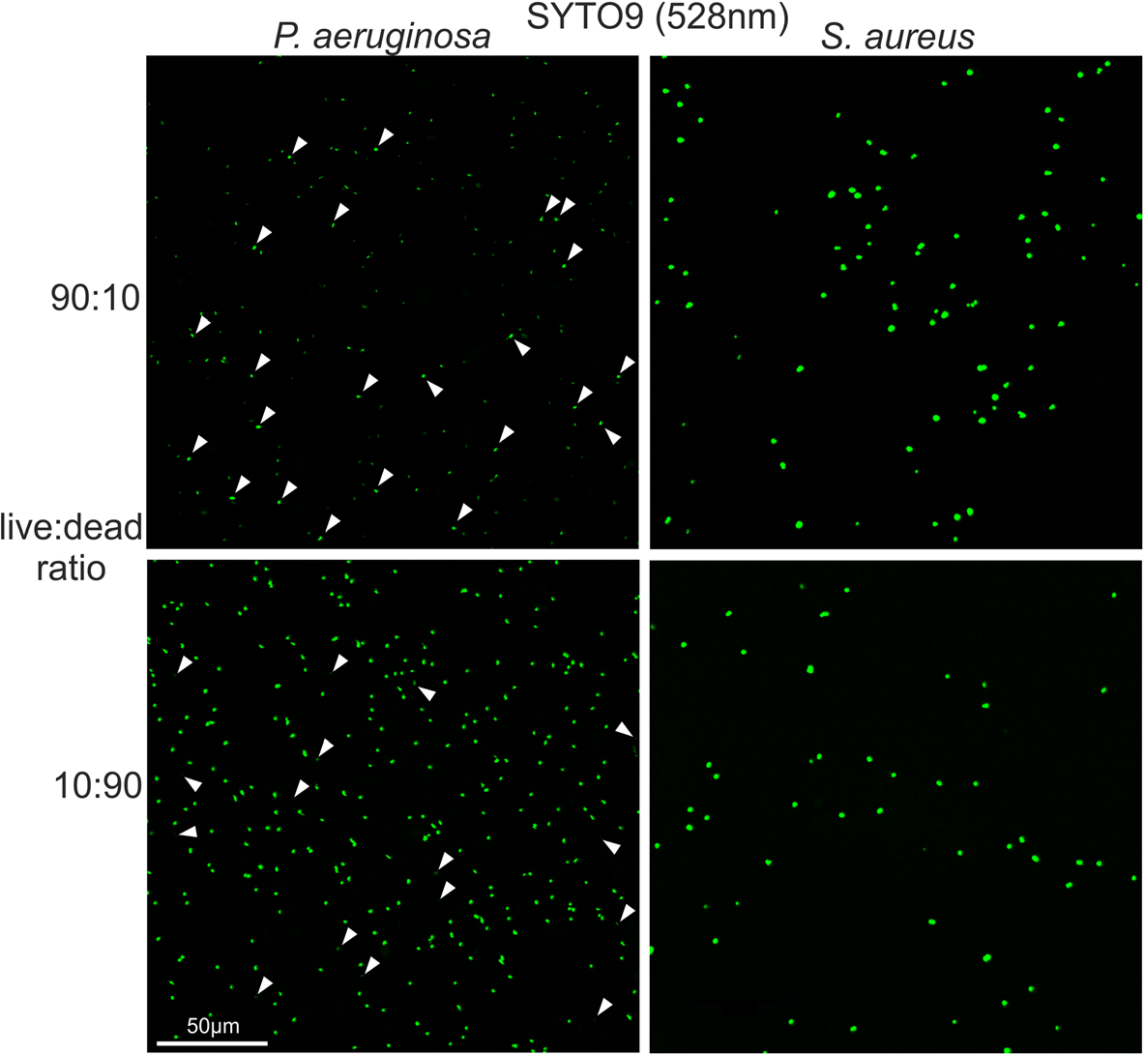
**SYTO9 staining analyzed with confocal microscopy.** Different live/dead proportions of *S. aureus* (right) and *P. aeruginosa* (left) cells were stained with SYTO9 and examined with CLSM. The live/dead ratios of 10:90 and 90:10 are shown for illustration and comparison. For *P. aeruginosa*, a small proportion of approximately 10% of the total stained cells exhibits weaker (in 10:90 ratio) or stronger (in 90:10 ratio) fluorescence compared with the rest of the cells. The cells having weaker or stronger fluorescence are indicated by arrowheads. For *S. aureus* no difference in SYTO9 signal intensity can be observed for live and dead cells.

**Figure 5:**
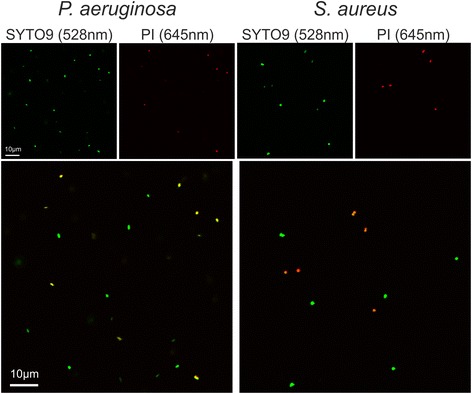
**SYTO9/PI staining analyzed with confocal microscopy.** Fluorescence images of the same samples at 528 nm (green) for SYTO9 signal, 645 nm (red) for PI signal and merged images are shown. 50:50 ratio of live and dead cells of *P. aeruginosa* (left) and *S. aureus* (right) was used. The cells were stained with SYTO9 and PI. Bacteria exhibiting red or yellow fluorescence are considered as dead cells.

Confocal laser scanning microscope (CLSM) was used to investigate individual cells stained with either SYTO9 alone or SYTO9/PI. The results gained from microscopy confirmed the data obtained with the microplate reader. Live and dead cells of *S. aureus* showed similar green fluorescence intensity when stained with SYTO9 alone, while live *P. aeruginosa* cells are stained clearly less than the dead ones (Figure 4, Additional file 3: Figure S3). Dead cells of both species, *S. aureus* and *P. aeruginosa*, exhibited red fluorescence after PI counterstaining (Figure 5). As expected, *S. aureus* cells that appear red after PI counterstaining show clearly weaker SYTO9 signal (Figure 5, Additional file 4: Figure S4). The mean integrated green fluorescence intensity was evaluated with CellProfiler software. It was found that dead *S. aureus* cells exhibited an almost 5-fold lower signal intensity compared to the living cells (Additional file 4: Figure S4d). Counterstaining of *P. aeruginosa* resulted in a strong reduction of SYTO9 fluorescence in dead cells. However, dead cells possessed much higher SYTO9 fluorescence than live cells before counterstaining. Therefore, the fluorescence reduction in dead cells after counterstaining only resulted in SYTO9 levels similar to that of living cells (Additional file 5: Figure S5).

## Discussion

The combined usage of SYTO9 and PI in a commercially available kit (*Bac*Light™ – Molecular Probes®) was first described in 1996 and is promoted as a rapid and reliable method for the assessment of bacterial viability that gives quantitative results and can be applied to microplate reader, flow cytometer and microscopes [7,20-22]. However, the reported data here revealed that there is a clear need for critical evaluation of results obtained from combined staining with SYTO9 and PI. Some of these factors have been described previously, mainly based on the results obtained from flow cytometric studies. For example, it has been reported that SYTO9 is not effective in staining some intact Gram-negative bacteria [9]. The same phenomenon was observed in the current study for the Gram-negative bacteria *P. aeruginosa* (Figure 1) and *E. coli* (Additional file 6: Figure S6). Most plausible explanation is that SYTO9 is not readily membrane permeable and has problems to cross the two cell membranes of Gram-negative bacteria. Another explanation might be that some bacterial cells actively export SYTO9 from their cytoplasm. It was also found that some cells exhibited yellow fluorescence instead of red after SYTO9 and PI staining, which is an often observed phenomenon when *Bac*Light™ kit is used [7]. The yellow fluorescence was generated when SYTO9 was not completely replaced by PI. Considering binding and releasing of SYTO9 and PI to/from nucleic acids are dynamic processes, it is possible that both the green and red dyes were retained within cells at the same time, indicating mostly dead cells [11].

### Critical factors influencing assessment of SYTO9/PI staining

#### Binding affinity of SYTO9 to live and dead cells

In the present study, we have observed that live Gram-negative bacteria are not always as easily accessible for SYTO9 staining. In our case, this resulted in an 18-fold stronger fluorescence signal for dead *P. aeruginosa* cells than for live ones. After counterstaining with PI the SYTO9 signal of dead cells was still slightly higher than that of living cells. Thus, live cells can be overestimated by combined staining. Viable cells might also be detected incorrectly as dead when membranes of viable cells can be perforated during cell division, cell wall synthesis, and injured during stress [23-26]. For example, Müsken and co-workers have reported that the isopropanol treated *P. aeruginosa* cells could not be properly assigned to live or dead cells with the microscope after staining with SYTO9 and PI [27]. This result was explained by an incomplete displacement of SYTO9 by PI in dead cells. Considering the finding in our study that intact *P. aeruginosa* cells are less efficiently stained by SYTO9 than dead cells, resulting in similar green fluorescence of both live and dead cells after counterstaining with PI, the results obtained by Müsken and co-workers could also be explained by the stronger SYTO9 signal of dead cells than the live ones. Since the combined SYTO9/PI staining was used in that study, it was not possible to assess the higher permeability of SYTO9 to dead cells. Thus, the knowledge obtained from single staining will help with interpreting such data: after subtracting the background and cross-signals, the cells appeared red fluorescence could be assigned as dead even if they possessed similar green fluorescence to the live cells.

### Bleaching of SYTO9

The fact of fast decrease of SYTO9 signal with time demands to take bleaching into consideration, especially for SYTO9/PI combined staining. One of the methods to determine the bleaching effect is to use replica in which NaCl solution or buffer as a control is added instead of PI. Thereby, the reduction in green fluorescence in the control can be subtracted from the samples where PI has been added before calculating the actual displacement of SYTO9 by PI. These values have to be determined empirically as they are highly dependent on the cell numbers, state of the cells and species.

### Background fluorescence

**Figure 6:**
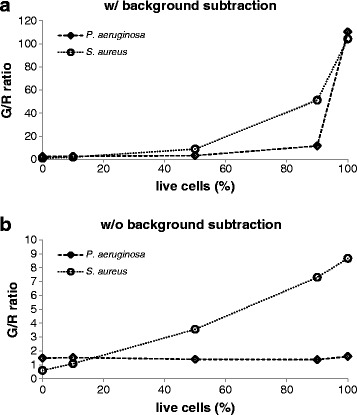
**Standard curves for determination of live/dead ratio.** Standard curves were generated according to the manufacturers’ instructions for the determination of live/dead ratios through dividing fluorescence intensity at 528 nm (G: green) by that at 645 nm (R: red), referred as G/R ratio. Values for different live/dead proportions of *S. aureus* and *P. aeruginosa* were plotted either with **(a)** or without **(b)** background subtraction.

Different background signals have to be taken into consideration when calculating the exact signal intensity to compare results from different conditions. First, the background of unbound dye has to be determined. The background of SYTO9 signal in NaCl solution is negligible compared to the strong signal of DNA-bound dye, but PI possesses a rather high fluorescence in the unbound form. Second, the emission signal intensity of one dye at the wavelength of the other dye should be considered. This background is referred as cross-signal. PI stained cells showed no signal at 528 nm (SYTO9 emission), whereas SYTO9 stained cells displayed rather high signal at 645 nm (PI emission) which cannot be neglected. For this purpose, the cross-signal of SYTO9 at 645 nm should be subtracted from the total signal obtained at 645 nm. The background signal can be obtained by measuring SYTO9 stained samples having different fluorescent intensities at 528 nm and plotting it against the signal at 645 nm (Additional file 7: Figure S7). The SYTO9 stained cells exhibited 2.7% of the 528 nm signal intensity at 645 nm channel. Regarding the overall much higher SYTO9 than PI signal this cross-signal can account for a substantial part of the 645 nm signal (Figure 3). Especially, if there are big differences in SYTO9 signal, this can bias the outcome, which is the case for *S. aureus* (Figure 3a) but less pronounced for *P. aeruginosa* (Figure 3b). Thus, it is recommended to perform the staining separately in order to minimize the cross-signal background. In practice, SYTO9/PI staining is often used to determine the killing efficiency of a substance against environmental samples with unknown amount of dead cells. Therefore, a standard curve for which the SYTO9 (green) to PI (red) fluorescence ratio (G/R ratio) is used to calculate the percentage of live/dead cells. Since a large proportion of the PI signal can come from the unbound dye, standard curves for evaluation with the microplate reader are only accurate after consideration of background fluorescence. Substantial differences are observed when generating standard curve with (Figure 6a) and without (Figure 6b) background subtraction. Due to much higher overall SYTO9 signal than PI signal the green/red ratio is not increasing linearly after subtracting the background (Figure 6a). In conclusion, for the 645 nm signal it is highly recommended to subtract background of unbound PI and cross-signal of SYTO9.

### Alternatives to Syto9

One alternative to SYTO9 for staining bacterial cells is SYBR green. It has similar properties regarding fluorescence enhancement upon binding and membrane permeability, and seems to have more homogeneous and reproducible pattern [9]. However, similar problems of stronger staining of dead Gram-negative bacteria [28] and dependency on the physiological state of cells [24] were reported. Other dyes often used to stain DNA, such as Acridine Orange, cannot be used for fast detection of total cell numbers in microplates due to required washing steps. They do not increase fluorescence signal intensity upon binding to DNA and unbound dye therefore needs to be washed off. Genetic engineering of bacterial strains with green fluorescent protein (GFP) instead of fluorescent dye is another useful tool for direct visualization of the cells [29,30]. However, the use of GFP has also several disadvantages. First, it cannot be used for environmental samples as the cells need to contain the gene encoding GFP. Second, the production of GFP might alter the cell metabolism and the expression of *gfp* is dependent on growth conditions and media. Furthermore, GFP is not necessarily disappearing from dead cells and thus influences the subsequent outcome by staining with PI.

## Conclusions

There are several critical factors in the use of viability staining of bacteria such as i) bleaching effects of SYTO9, ii) different binding affinities of SYTO9 to live and dead cells and iii) background fluorescence and cross-signal of one dye into another dye’s channel. Nevertheless, using appropriate controls, the combination of SYTO9 and PI can be a very useful tool to detect the live and dead cells in regard to membrane integrity, and for example enables high throughput screening for toxic substances in microtiter plates. For a proper evaluation background controls have to be subtracted, bleaching of SYTO9 has to be considered, and differences in SYTO9 staining for live/dead cells of Gram-negative bacteria have to be taken into account.

## Methods

### Chemicals and reagents

Chemicals and reagents were purchased from Sigma Aldrich (Switzerland) if not mentioned elsewise.

### Bacterial strains and growth conditions

Bacterial strains were obtained from ‘The Leibniz Institute DSMZ - German Collection of Microorganisms and Cell Cultures GmbH’. *Pseudomonas aeruginosa* (DSM No. 1117) and *Staphylococcus aureus* (DSM No. 20231) were grown on Tryptic Soy Agar at 37°C. The cells from agar plates were used to inoculate 50 mL liquid culture in a 1 L shake flask containing 30% Tryptic Soy Broth supplemented with 0.25% glucose. The culture was incubated at 37°C and 160 rpm for overnight (about 16 hours).

### Preparation of live and dead bacterial cells

Both *P. aeruginosa* and *S. aureus* cells obtained from overnight cultures were at the end of the exponential growth phase and the beginning of the stationary phase, and thus used for preparation of live and dead cells. The cultures (50 mL) were centrifuged (7000 *g*, 10 min, 22°C) and the pellet was resuspended in 1 mL 0.9% NaCl solution. 0.5 mL of the cell suspension was added to 20 mL of 70% isopropanol to obtain dead cells and 0.5 mL to 20 mL of 0.9% NaCl to obtain live cells. Cells were incubated for 1 hour at room temperature before being spun down and washed with 0.9% NaCl once. OD_595_ was adjusted to 1.2 for *P. aeruginosa* with a total cell number of 2.0 × 10^9^ cells per mL and 2.5 for *S. aureus* with a total cell number of 2.0 × 10^8^ cells per mL. Further dilution was done to reach indicated ODs. Mixtures of different proportions of live/dead cells were prepared prior to staining.

Viable cell numbers were determined by spotting 5 μl of a 1:5 dilution series on Tryptic Soy Agar.

### Microplate reader

100 μl of cell suspension were added per well of a black 96-well plate (BRAND*plates*® pureGrade™). 50 μl of 2.5 μM SYTO9 (S-34854, Molecular Probes®) was added per well before incubating on the orbital shaker for 15 minutes in the dark. Fluorescence intensity was measured with a Synergy HT Multi-Detection Microplate Reader (BioTek®) using a 488/20 nm excitation filter (for both SYTO9 and PI) and a 528/20 nm (SYTO9 emission wavelength) and 645/40 nm (PI emission wavelength) emission filter. 50 μl of 15 μM propidium iodide was added per well before incubating additional 15 minutes on the orbital shaker in the dark and measuring fluorescence intensity with the same filter sets.

### Confocal laser scanning microscope (CLSM)

Cells were treated and stained in the same way as for the evaluation with the microplate reader. Zeiss Axioplan 2 with LSM 510 Scanning Module and 40x magnification objective was used to analyze specimens. The laser was used at 488 nm for excitation, and the emission was observed at 528 nm (SYTO9) and 645 nm (PI). Zeiss ZEN software was used to acquire images and CellProfiler software was used to analyze signal intensities (http://www.cellprofiler.org/).

## Additional files

Additional file 1: Figure S1. SYTO9/PI staining analyzed with confocal microscopy. 50:50 ratios of live/dead *S. aureus* (a) and *P. aeruginosa* (b) cells stained with SYTO9 and PI examined with CLSM. Merged fluorescence images of the same sample at 528 nm (green) for SYTO9 signal and 645 nm (red) for PI signal are shown. Half of the cells appear red/yellow (dead cells) while the rest is green (live cells). The numbers of the live and dead cells are comparable and the cells have a similar shape for the same species. Additional file 2: Figure S2. Bleaching of SYTO9 over time. Different amount of live or dead cells of *S. aureus* (S.a.) and *P. aeruginosa* (P.a.) were stained with SYTO9, respectively. After 15 min incubation fluorescence intensity at 528 nm was automatically measured every 5 minutes with the microplate reader. Relative fluorescence intensity is plotted against time. Additional file 3: Figure S3. Images of SYTO9 stained *P. aeruginosa* analyzed with CellProfiler software. *P. aeruginosa* cells having live/dead ratios of 10:90 (left) and 90:10 (right) were stained with SYTO9, examined with the CLSM and analyzed by CellProfiler software. The original images are shown in (a). Single cells identified by the software are shown in (b). The integrated intensities of the identified cells are shown in (c). The intensities are summarized in a histogram (d), which corresponds nicely to the used ratios when an intensity threshold of 60 is assumed to differentiate live and dead. Additional file 4: Figure S4. Images of SYTO9/PI stained *S. aureus* analyzed with CellProfiler software. *S. aureus* cells with a live/dead ratio of 50:50 were stained with SYTO9 and PI. Images were taken with the CLSM and analyzed by CellProfiler software. The original image (a) of the same area is shown for the SYTO9 (right) and PI (left) fluorescence. Single cells were identified by the software in both fluorescence channels (b). The integrated intensities of SYTO9 fluorescence were calculated either for dead cells (identified by PI fluorescence, 27 cells identified) or for live cells (identified by SYTO9 fluorescence excluding PI fluorescent areas, 25 cells identified) (c). The intensities are summarized in histograms (d). Additional file 5: Figure S5. Images of SYTO9/PI stained *P. aeruginosa* analyzed with CellProfiler software. *P. aeruginosa* cells with a live/dead ratio of 50:50 were stained with SYTO9 and PI. Images were taken with the CLSM and analyzed by CellProfiler software. The original image (a) of the same area is shown for the SYTO9 (right) and PI (left) fluorescence. Single cells were identified by the software in both fluorescence channels (b). The integrated intensities of SYTO9 fluorescence were calculated either for dead cells (identified by PI fluorescence, 66 cells identified) or for live cells (identified by SYTO9 fluorescence excluding PI fluorescent areas, 77 cells identified) (c). The intensities are summarized in histograms (d). Additional file 6: Figure S6. SYTO9 staining analyzed with microplate reader. Relative fluorescence intensity at 528 nm is shown for different live/dead proportions of *E. coli*. Values were measured after staining with SYTO9 for 15 minutes (green diamonds) and after additional 15 minutes counterstaining with PI (red circles). Cell optical densities (OD_595_) of 0.12 was used. Error bars represent 3 individual repeats with 3 replicas. Additional file 7: Figure S7. SYTO9 cross-signal at 645 nm. Fluorescence intensities of different samples stained with SYTO9 alone were measured at 528 nm and 645 nm. The two relative fluorescence intensities were plotted against each other to calculate the mean cross-signal of SYTO9 at 645 nm by a linear regression.

## Abbreviations

PI: Propidium iodide
NaCl: Sodium chloride
OD: Optical density
CLSM: Confocal laser scanning microscope
RFU: Relative fluorescence intensity unit
GFP: Green fluorescent protein
