# Supplementary figures and images for: Critical aspects of using bacterial cell viability assays with the fluorophores SYTO9 and propidium iodide
Source: BMC Microbiol. 2015 Feb 18;15:36. doi: 10.1186/s12866-015-0376-x (PMC4337318; doi:10.1186/s12866-015-0376-x)

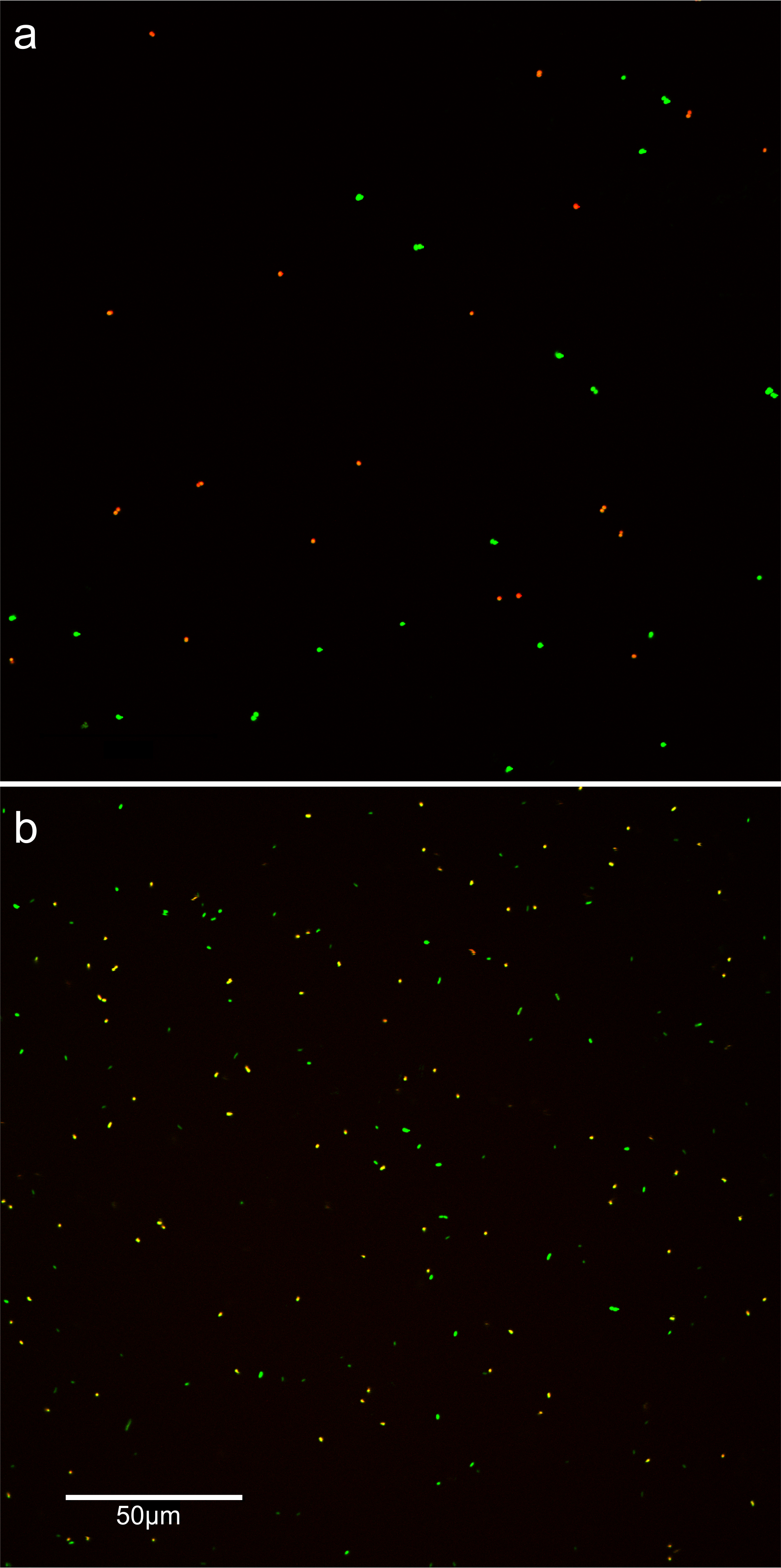

Supplement: Additional file 1: Figure S1. — SYTO9/PI staining analyzed with confocal microscopy. 50:50 ratios of live/dead S. aureus (a) and P. aeruginosa (b) cells stained with SYTO9 and PI examined with CLSM. Merged fluorescence images of the same sample at 528 nm (green) for SYTO9 signal and 645 nm (red) for PI signal are shown. Half of the cells appear red/yellow (dead cells) while the rest is green (live cells). The numbers of the live and dead cells are comparable and the cells have a similar shape for the same species. [file 12866_2015_376_MOESM1_ESM.png]

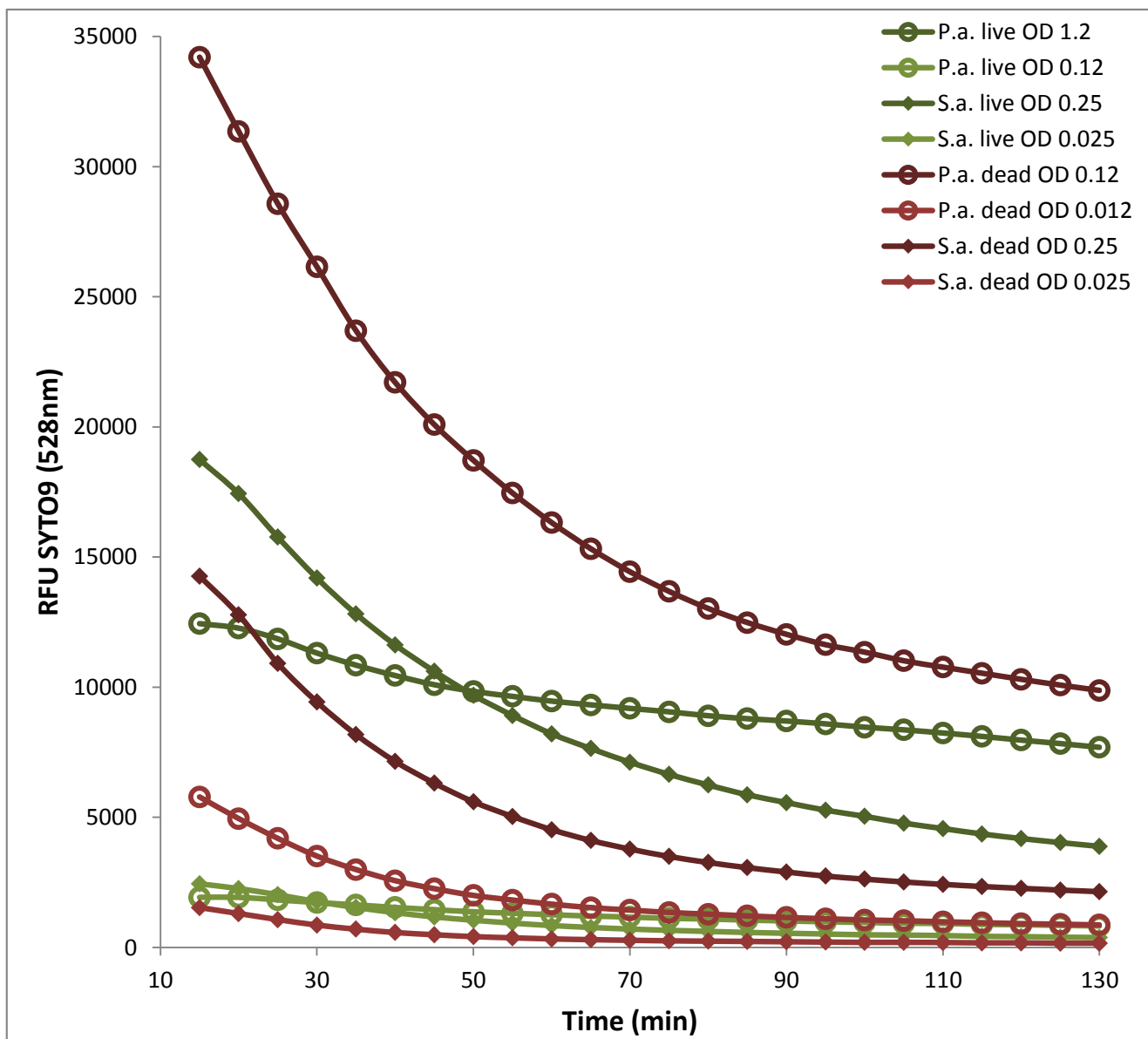

Supplement: Additional file 2: Figure S2. — Bleaching of SYTO9 over time. Different amount of live or dead cells of S. aureus (S.a.) and P. aeruginosa (P.a.) were stained with SYTO9, respectively. After 15 min incubation fluorescence intensity at 528 nm was automatically measured every 5 minutes with the microplate reader. Relative fluorescence intensity is plotted against time. [file 12866_2015_376_MOESM2_ESM.pdf]

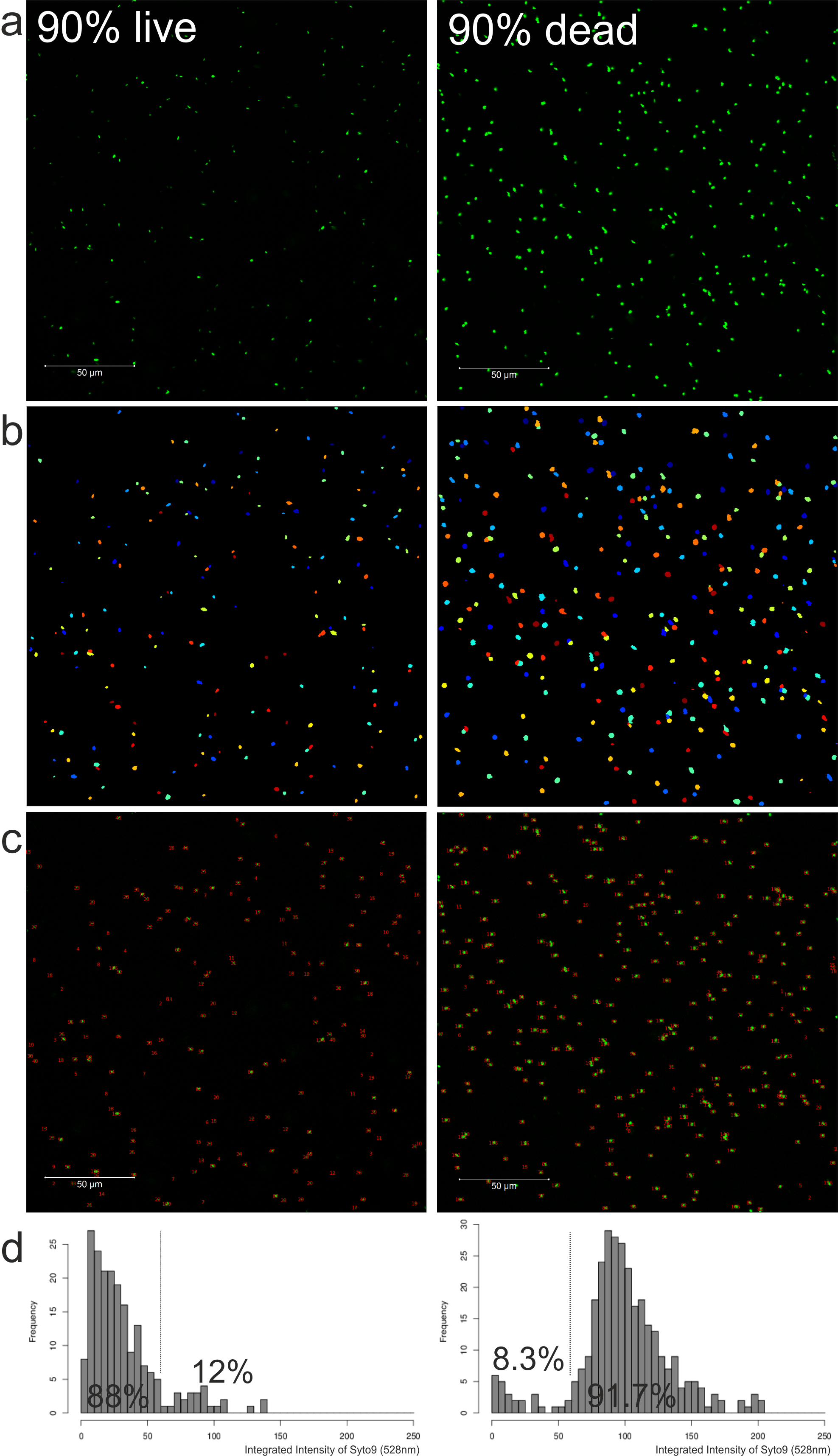

Supplement: Additional file 3: Figure S3. — Images of SYTO9 stained P. aeruginosa analyzed with CellProfiler software. P. aeruginosa cells having live/dead ratios of 10:90 (left) and 90:10 (right) were stained with SYTO9, examined with the CLSM and analyzed by CellProfiler software. The original images are shown in (a). Single cells identified by the software are shown in (b). The integrated intensities of the identified cells are shown in (c). The intensities are summarized in a histogram (d), which corresponds nicely to the used ratios when an intensity threshold of 60 is assumed to differentiate live and dead. [file 12866_2015_376_MOESM3_ESM.png]

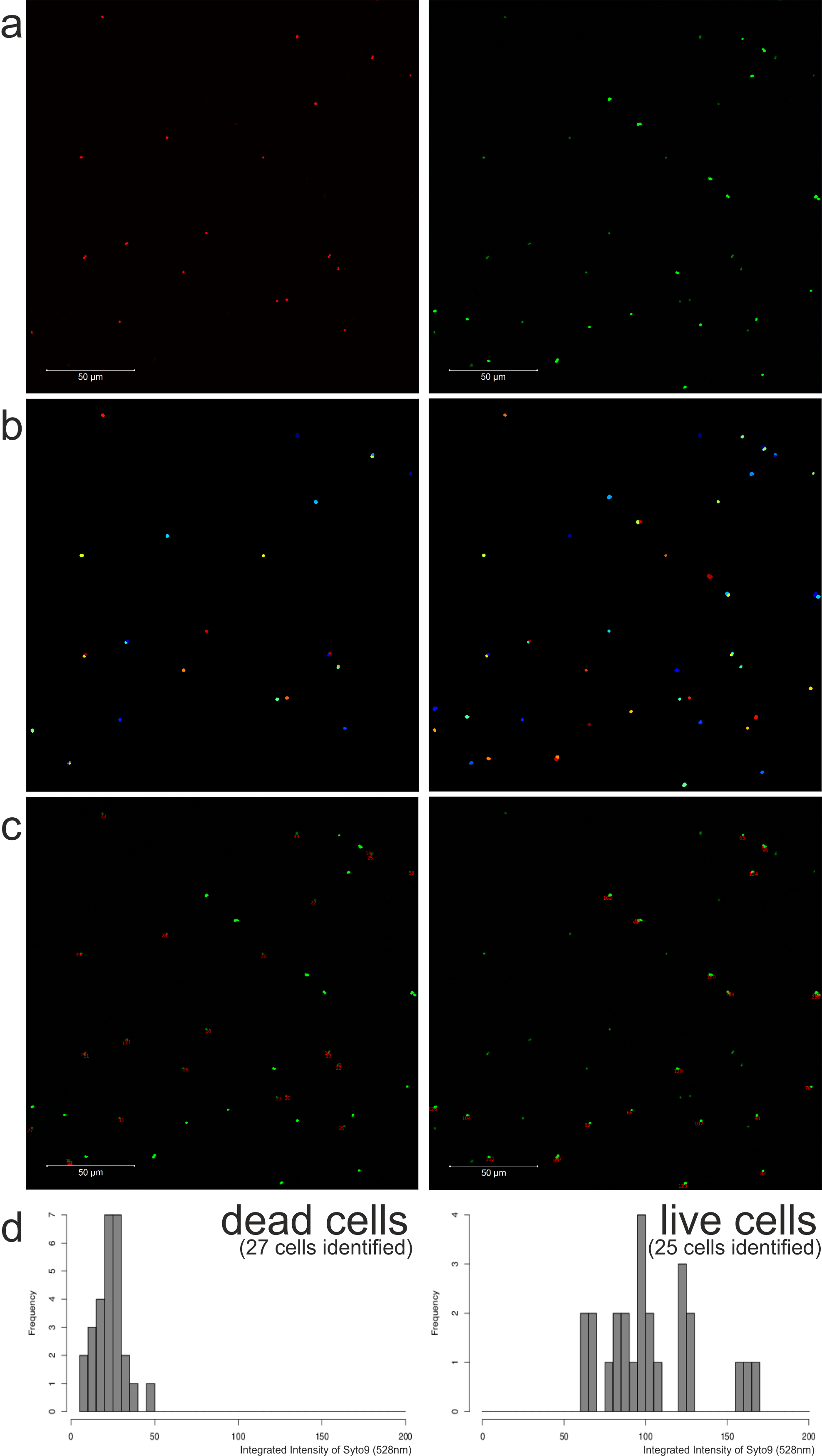

Supplement: Additional file 4: Figure S4. — Images of SYTO9/PI stained S. aureus analyzed with CellProfiler software. S. aureus cells with a live/dead ratio of 50:50 were stained with SYTO9 and PI. Images were taken with the CLSM and analyzed by CellProfiler software. The original image (a) of the same area is shown for the SYTO9 (right) and PI (left) fluorescence. Single cells were identified by the software in both fluorescence channels (b). The integrated intensities of SYTO9 fluorescence were calculated either for dead cells (identified by PI fluorescence, 27 cells identified) or for live cells (identified by SYTO9 fluorescence excluding PI fluorescent areas, 25 cells identified) (c). The intensities are summarized in histograms (d). [file 12866_2015_376_MOESM4_ESM.png]

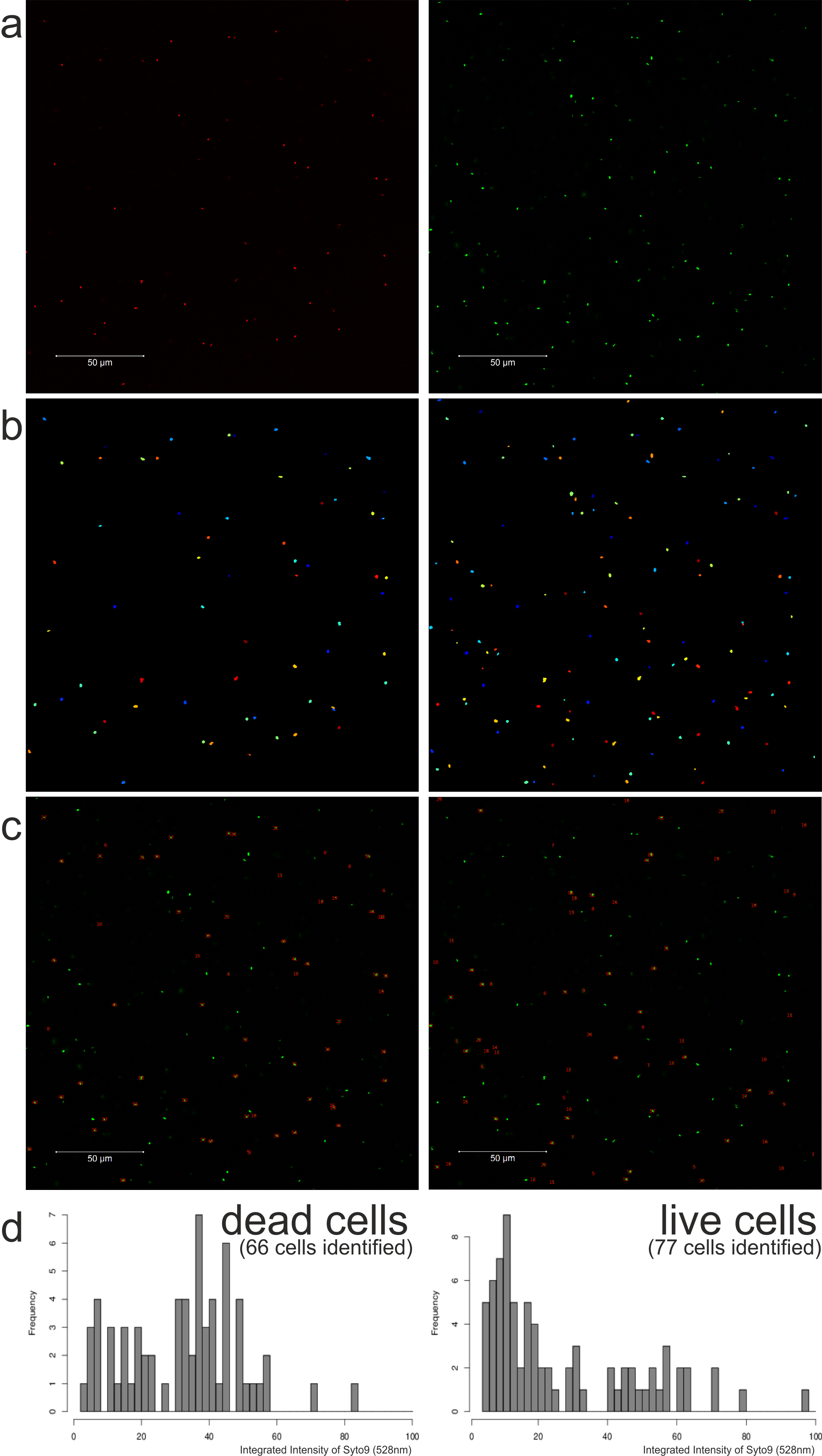

Supplement: Additional file 5: Figure S5. — Images of SYTO9/PI stained P. aeruginosa analyzed with CellProfiler software. P. aeruginosa cells with a live/dead ratio of 50:50 were stained with SYTO9 and PI. Images were taken with the CLSM and analyzed by CellProfiler software. The original image (a) of the same area is shown for the SYTO9 (right) and PI (left) fluorescence. Single cells were identified by the software in both fluorescence channels (b). The integrated intensities of SYTO9 fluorescence were calculated either for dead cells (identified by PI fluorescence, 66 cells identified) or for live cells (identified by SYTO9 fluorescence excluding PI fluorescent areas, 77 cells identified) (c). The intensities are summarized in histograms (d). [file 12866_2015_376_MOESM5_ESM.png]

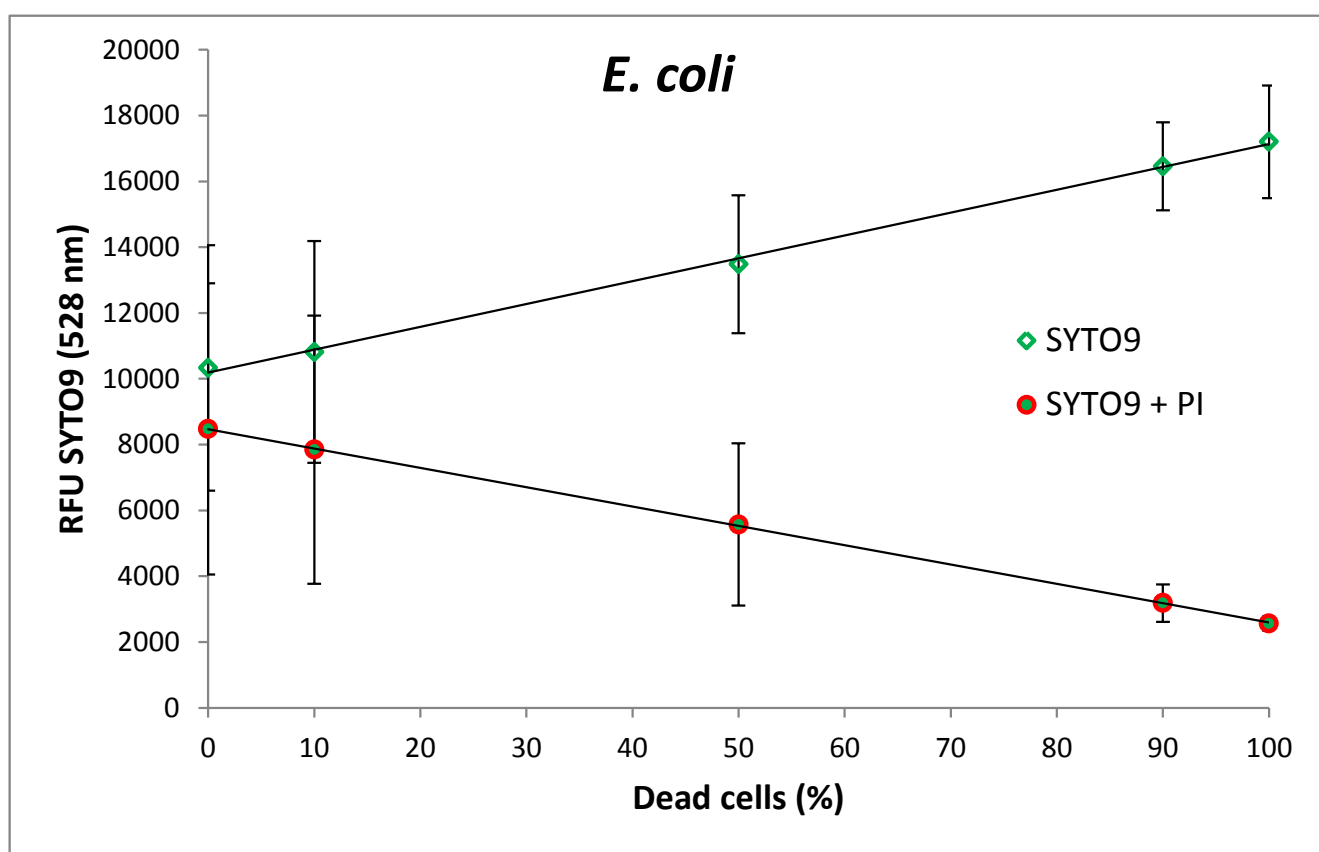

Supplement: Additional file 6: Figure S6. — SYTO9 staining analyzed with microplate reader. Relative fluorescence intensity at 528 nm is shown for different live/dead proportions of E. coli. Values were measured after staining with SYTO9 for 15 minutes (green diamonds) and after additional 15 minutes counterstaining with PI (red circles). Cell optical densities (OD595) of 0.12 was used. Error bars represent 3 individual repeats with 3 replicas. [file 12866_2015_376_MOESM6_ESM.pdf]

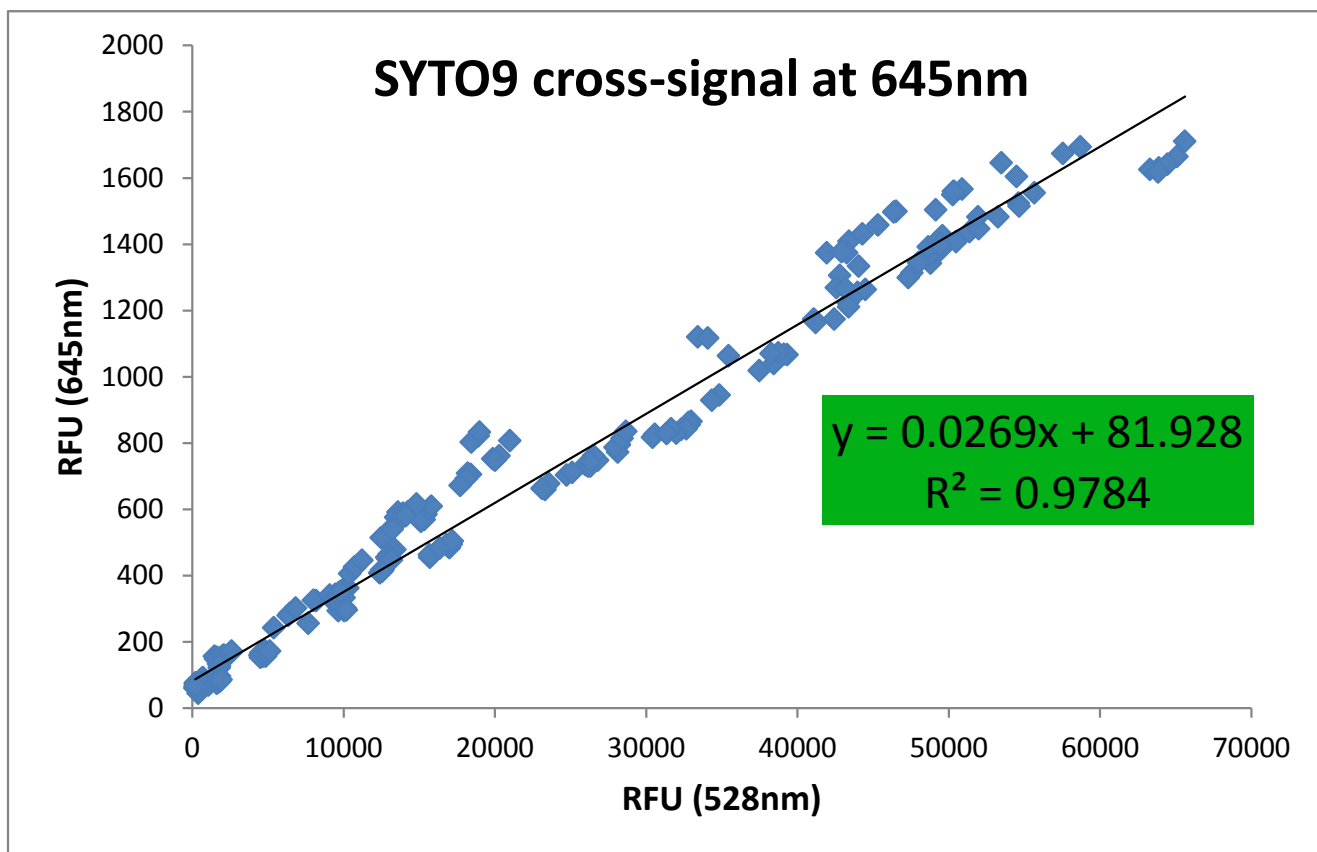

Supplement: Additional file 7: Figure S7. — SYTO9 cross-signal at 645 nm. Fluorescence intensities of different samples stained with SYTO9 alone were measured at 528 nm and 645 nm. The two relative fluorescence intensities were plotted against each other to calculate the mean cross-signal of SYTO9 at 645 nm by a linear regression. [file 12866_2015_376_MOESM7_ESM.pdf]
